# Rapamycin slows down gut aging

**DOI:** 10.18632/aging.100963

**Published:** 2016-05-12

**Authors:** Uma Gaur, Xiaolan Fan, Mingyao Yang

**Affiliations:** Institute of Animal Genetics and Breeding, Sichuan Agricultural University, Chengdu, Sichuan, China

**Keywords:** rapamycin, gut homeostasis, intestinal stem cell, Drosophila

The last decade has witnessed an exponential increase in the researches being carried out to combat aging using drug therapy. Few specific drugs like Metformin, Rapamycin and Resveratrol have displayed promising results in anti-aging research of mice, fruit fly and worm. Basically these drugs interfere genetically with the important aging pathways such as IIS, TOR, and NAD+ dependent pathway, and results in lifespan extension in a number of species ranging from worm to mammals [1].

Rapamycin which was originally an immuno-suppressive drug used for kidney transplant patients for years, is an inhibitor of protein kinase target of rapamycin (TOR). Rapamycin inhibits the TOR by forming a complex with FKBP12, which goes on to bind with TOR complex1. The mechanism of rapamycin mediated lifespan extension has largely remained conserved in different model species, for example the genetic mutations/deletions of S6K and 4E-BP in mice and its homologues in worms have resulted in extended lifespan. Rapamycin treatment has led to reduced phosphorylation of S6K in *Drosophila* resulting in lifespan extension along with increased stress resistance, reduced fecundity and increased lipid levels. Also rapamycin treatment has shown to further extend the lifespan of some long lived IIS mutants, and dietary restricted flies, indicating additional mechanisms of lifespan extension [2].

Gut has been considered as an unique important target organ in mediating the lifespan extension at the organismal level because of its immunity and nutrition intake [3]. Also the gut equanimity is very important to maintain overall body health during aging in *Drosophila*. In flies the midgut stability is maintained by multipotent intestinal stem cells (ISCs) in hindgut proliferation zone (HPZ) and controlled by locally emanating Wingless (Wg, a *Drosophila* Wnt homologue) and Hh signals. Under the stress conditions such as aging, pathogen exposure, genotoxins or ROS inducing compounds, the ISC proliferation increases strongly in order to restore the large parts of intestinal epithelium. The epithelial integrity is disrupted by this regenerative event leading to accumulation of mis-differentiated cells and resulting in a dysplastic pheno-type [4]. Interestingly enough this dysplastic phenotype has been shown to be more prominent in female guts. Recent study showed that in female flies gut the stem cells divide more often and form small tumors resulting in more deteriorated gut in grown up females than males. This study explored the sex specific pathologies of the aging gut, which is an important yet unanswered question, as why gastrointestinal diseases and cancers are gender biased [5].

**Figure 1:**
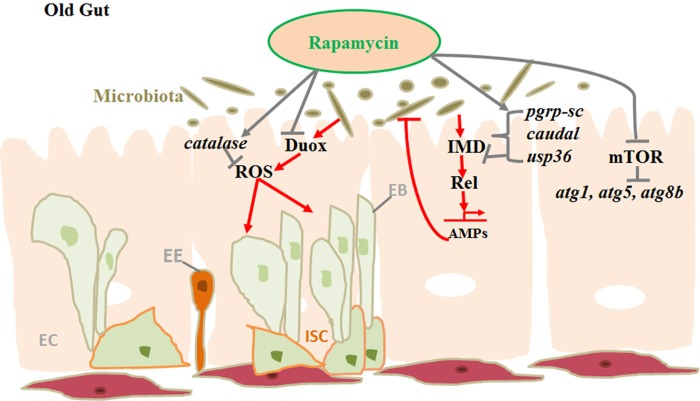
Model displaying gut homeostasis by rapamycin treatment in aging gut.

In dysplastic guts the disrupted gut barrier leads to the increased movement of the microbial products and activates the mucosal immune system secreting the inflammatory mediators, which might increase the barrier dysfunction further [6]. The lifespan extending properties of rapamycin has been studied in detail in various model species, but its effect on gut stability remains unexplored. In order to understand how rapamycin may affect the homeostasis in aging guts, we have used *Drosophila* as a model system to carry out such study [7]. Our results established that rapamycin can slow down the ISC proliferation rate and intestinal barrier dysfunction in the aging guts and induce autophagy in *Drosophila* intestinal epithelium. We have proposed that, along with inhibiting mTOR, rapamycin can also significantly delay the microbial expansion by upregulating the negative regulators of IMD/Rel pathway in the aging guts (Figure 1).

The expression level of catalase gene, which is responsible for protection against oxidative damage by reactive oxygen species (ROS), is significantly up regulated in rapamycin treated flies suggesting that rapamycin can improve the intestinal capacity for antioxidants. The autophagy genes atg1, atg5 and atg8b displayed increased mRNA expression level in intestinal tissues, indicating that rapamycin can induce autophagy and increase the lifespan. The anti-microbial peptide (AMP) gene dpt and the dual oxidase gene (duox), which is associated with reactive oxygen species (ROS) levels, were significantly decreased in rapamycin treated aged guts, indicating that rapamycin may reduce the intestinal ROS accumulation in aging flies [7]. Thus rapamycin can slow down ISC proliferation along with inhibiting mTOR, thereby delaying the gut aging and promoting the lifespan extension.

These findings clearly show that rapamycin treatment maintains the gut stability during aging in Drosophila, leading to healthspan and lifespan extension. It should be noted that due to the high evolutionary conservation of mTOR pathway in different species, the same mechanism underlying rapamycin treatment could apply in other species as well. Thus rapamycin holds great future potential in various gastrointestinal disorders and cancers along with lifespan extension.
